# Occupational exposures and kidney cancer among 25 000 male offshore petroleum industry workers: relative risks and healthy worker survivor bias

**DOI:** 10.1093/aje/kwaf039

**Published:** 2025-02-28

**Authors:** Nita K Shala, Marit B Veierød, Ronnie Babigumira, Leon A M Berge, Sven O Samuelsen, Jorunn Kirkeleit, Magne Bråtveit, Melissa C Friesen, Alexander P Keil, Debra T Silverman, Nathaniel Rothman, Lan Qing, Jo S Stenehjem, Tom K Grimsrud

**Affiliations:** Department of Research, Cancer Registry of Norway, Norwegian Institute of Public Health, Oslo, Norway; Oslo Centre for Biostatistics and Epidemiology, Department of Biostatistics, Institute of Basic Medical Sciences, University of Oslo, Oslo, Norway; Oslo Centre for Biostatistics and Epidemiology, Department of Biostatistics, Institute of Basic Medical Sciences, University of Oslo, Oslo, Norway; Department of Research, Cancer Registry of Norway, Norwegian Institute of Public Health, Oslo, Norway; Oslo Centre for Biostatistics and Epidemiology, Department of Biostatistics, Institute of Basic Medical Sciences, University of Oslo, Oslo, Norway; Department of Research, Cancer Registry of Norway, Norwegian Institute of Public Health, Oslo, Norway; Oslo Centre for Biostatistics and Epidemiology, Department of Biostatistics, Institute of Basic Medical Sciences, University of Oslo, Oslo, Norway; Department of Mathematics, University of Oslo, Oslo, Norway; Department of Occupational Medicine and Epidemiology, National Institute of Occupational Health, Oslo, Norway; Department of Global Public Health and Primary Care, University of Bergen, Bergen, Norway; Division of Cancer Epidemiology and Genetics, Occupational and Environmental Epidemiology Branch, National Cancer Institute, Bethesda, MD, United States; Division of Cancer Epidemiology and Genetics, Occupational and Environmental Epidemiology Branch, National Cancer Institute, Bethesda, MD, United States; Division of Cancer Epidemiology and Genetics, Occupational and Environmental Epidemiology Branch, National Cancer Institute, Bethesda, MD, United States; Division of Cancer Epidemiology and Genetics, Occupational and Environmental Epidemiology Branch, National Cancer Institute, Bethesda, MD, United States; Division of Cancer Epidemiology and Genetics, Occupational and Environmental Epidemiology Branch, National Cancer Institute, Bethesda, MD, United States; Department of Research, Cancer Registry of Norway, Norwegian Institute of Public Health, Oslo, Norway; Oslo Centre for Biostatistics and Epidemiology, Department of Biostatistics, Institute of Basic Medical Sciences, University of Oslo, Oslo, Norway; Department of Research, Cancer Registry of Norway, Norwegian Institute of Public Health, Oslo, Norway

**Keywords:** kidney cancer, petroleum industry, hydrocarbon exposure, time-varying confounding, healthy worker survivor bias

## Abstract

Kidney cancer has been a suspected occupational disease in petroleum workers. Health conditions that are linked to kidney cancer may prompt termination or change of work and thereby restrict occupational exposures in high-risk individuals, creating a healthy worker survivor bias (HWSB). We examined associations between occupational exposures and kidney cancer among males in the Norwegian offshore petroleum workers cohort using a case-cohort design, with 169 incident cancers identified by linkage to national registry data (1999-2021) and a subcohort of 2090 noncases, all employed between 1965 and 1998. Relative risks (hazard ratios [HRs]) by cumulative exposure to benzene, crude oil, chlorinated degreasing agents (CDA), asbestos, welding fumes, or surface treatment (priming, painting) were estimated by weighted Cox regression. Inverse exposure-response trends suggested HWSB, reinforced by analyses of necessary components of HWSB. Bias was partly alleviated by adjustment for total employment duration and by 20-year lagging of cumulative exposure to benzene, crude oil, or CDA. Workers in surface treatment (ever vs never) showed increased HR = 2.22, 95% confidence interval [CI], 1.04-4.72 (9 cases, only). For asbestos and welding fumes, the initial inverse trends largely remained after adjustment. In sum, we could neither confirm nor exclude an occupational impact on kidney cancer.

## Introduction

It has been estimated that 50% of all incident kidney cancers remain unexplained after accounting for smoking, obesity, diabetes, and hypertension.^1^^,^^2^ Two environmental agents are recognized as human kidney carcinogens (Group 1) by the International Agency for Research on Cancer (IARC): trichloroethylene (TCE) and ionizing radiation. Limited evidence remains for other IARC Group 1 carcinogens, such as welding fumes, arsenic, cadmium compounds, and perfluorooctanoic acid.^3-5^

Kidney cancer in petroleum workers was investigated and discussed in the 1980s and 1990s.^6^^,^^7^ Increased mortality from kidney cancer was reported among UK petroleum distribution workers, particularly drivers.^8^^,^^9^ A large international population-based case-control study provided evidence of excess risk following exposure to coal and tar products, gasoline and other petroleum products, as well as dry-cleaning products and asbestos.^10^ A 1997 review pointed to the need for taking healthy worker bias into account, and was unable to conclude on a causal effect from gasoline.^11^ Subsequent studies have reported increased risks among oil refinery workers,^12^^,^^13^ gasoline service station workers,^14^ and oil tanker drivers.^15^ Complex exposure patterns are common in the petroleum industry, with simultaneous or sequential exposures to multiple single or mixed agents.^16-18^ Moreover, a healthy hire bias and a healthy worker survivor bias (HWSB) still may represent challenges for the risk analyses.^19^^,^^20^

A 2021 systematic review and meta-analysis of cohort and case-control studies among petroleum workers did not suggest an occupational kidney cancer risk, calling for better exposure data and confounder control.^21^ A more recent meta-analysis of workers exposed to benzene suggested 17%-25% increased risk of kidney cancer in North America and Europe.^22^

In the Norwegian offshore petroleum workers (NOPW) cohort, we recently reported associations between exposure to benzene or accompanying hydrocarbons and cancer of the bladder, lung, skin, and lymphohematopoietic tissue system.^23-26^ Benzene metabolites are carcinogenic; they follow the circulation and are excreted in the urine, and an effect from benzene on kidney cancer could possibly explain the observations among workers in the petroleum industry. In the NOPW cohort, workers have also been exposed to chlorinated degreasing agents (CDA), including dichloromethane, TCE, 1,1,1-trichloroethane, and tetrachloroethylene, used for cleaning metal parts and surfaces, and for dry cleaning.^17^ Further, drilling and maintenance tasks have entailed exposures to asbestos, welding fumes, and crystalline silica, which all have been suspected kidney carcinogens.^1^^,^^27^ We aimed to investigate the associations between these occupational exposures and kidney cancer in a prospective study in the NOPW cohort, using industry-specific job-exposure matrices (JEMs). Drawing on earlier reviews,^11^^,^^28^ we also aimed to investigate the potential of HWSB.

## Methods

### Cohort, case identification, and study sample

The NOPW cohort includes 25 347 male and 2570 female workers who worked ≥20 days at oil platforms on the Norwegian continental shelf between 1965 and 1998, as reported on questionnaires in 1998 (hereafter baseline). Details on the establishment of this survey-based cohort have been presented elsewhere.^29^

The cohort was linked to the National Population Register for information on emigration and vital status and to the Cancer Registry of Norway (CRN) for incident cancers, using the unique 11-digit personal identity numbers assigned to all residents in Norway since 1960.^30^ The degree of completeness and validity of the CRN is highly secured by reporting from several sources,^31^ and 82% of all kidney cancers were morphologically verified in 2001-2005, and 92% in 2017-2021.^30^^,^^32^

Kidney cancers were defined as incident primary tumors of the renal parenchyma (Code C64 in International Classification of Diseases [ICD] 10th revision), diagnosed from July 1, 1999 (start of follow-up) through 2021 (end of follow-up). Women were excluded from this study as <10 cases occurred during follow-up. Histological subtypes of kidney cancer were categorized according to the ICD for Oncology Third revision (ICD-O-3, Table S1).

At baseline, each worker reported up to 8 offshore employments,^29^ which were assigned to 27 job categories. Overlapping employment records were harmonized according to a previously described method.^33^ The first and last employment were electronically recorded for all cohort members, but intermediate employments had to be manually extracted. For cost-efficiency reasons, we opted for a stratified case-cohort design, where the manual extraction of work history data was done only for all cases and a subcohort drawn randomly within strata of 5-year birth cohorts, and retrospectively confirmed as noncases.^34^ The final study sample consisted of 2259 male workers, including 169 incident kidney cancer cases and 2090 noncases (Figure S1).

### Assessment of exposures

#### Chlorinated degreasing agents, crude oil, welding fumes, and surface treatment work

In 2005, experts in offshore industrial hygiene developed probability-based JEMs for known and suspected carcinogens relevant for the 27 job categories in the NOPW cohort.^17^^,^^35-37^

Assessments of exposure to CDA (skin/inhalatory), crude oil (skin exposure), and welding fumes (inhalatory) were based on job-category- and time-period-specific ratings (JEM-scores) by experts considering probability, frequency, and intensity of the exposures: 0 = improbable exposure; 1 = possible exposure; 2 = probable exposure (at least 50% exposed workers within the job category); and 3 = the highest ranking of probable exposure. Three time periods were assessed: before 1980, 1980-1989, and 1990-1998.

The kidney carcinogen TCE was used in laboratories and as a cleaning and degreasing detergent for several purposes. Occupational use of TCE ceased around 1985-1989 because of its toxic effects, but no specific measurements or documentation exist on the amounts or frequency of use. Hence, the available JEM does not estimate TCE exposures specifically but represents exposure to CDA as a broad group of chlorinated solvents.^35^

Work in the job category surface treatment (priming and painting) was considered to represent a potentially combined exposure to lead, crystalline silica, paint, and solvents including CDAs. Duration of exposure to surface treatment was divided into unlikely exposed (no work reported in the job category) or exposed below or exposed above the median duration of employment with surface treatment.

#### Benzene exposure

The benzene JEM was developed with a task-oriented approach resulting in semiquantitative benzene exposure intensity ratings (JEM-scores).^38^ The assessment was based on monitoring data (where available) and detailed information on 9 job-specific tasks involving benzene, according to previously described methods,^38^^,^^39^ for 27 job categories and the calendar periods <1980, 1980-1989, and 1990-1998. The relative contribution from inhalation and dermal absorption was not estimated. For the present study, 2 minor modifications of the JEM were implemented: 6 undetermined JEM-score values were assigned a score of 0.05, and process technicians were merged and assigned the mean JEM-score for the 2 original subgroups.

The benzene JEM-scores were recalculated into proportionally corresponding parts per million (ppm) values in the work atmosphere, by a method slightly modified from Stenehjem et al.,^26^ with an average level of 0.036 ppm corresponding to a JEM-score of 1.35.

#### Asbestos exposure

Exposure estimates for asbestos were prepared with the same task-oriented approach as that described for benzene.^38^ No quantitative exposure estimates were assigned, leaving semiquantitative ratings of asbestos intensity (JEM-scores) across 27 job categories for the periods <1985 and 1985-1998.^37^

#### Exposure metrics

For all exposure agents, duration of exposure was defined as the number of years up to baseline in job categories with a JEM-score >0. Cumulative exposure was defined as the sum of products of JEM-score multiplied with duration, summing values for all relevant employment periods in each individual work history through 1998. The average intensity of exposure was derived by dividing the cumulative exposure by duration of exposure. The cumulative and average intensity metrics were categorized as unlikely exposed (JEM-score = 0 in all employments), and tertiles among exposed cases and noncases. For average benzene intensity, we used cut points according to the rating of job categories by Bråtveit et al.^38^: >0.000-0.015, >0.015-0.030, and >0.030-0.057 ppm.

#### Other variables

Total employment duration offshore (years) was derived from individually reported active employment in the petroleum industry 1965-1998. Body mass index (BMI, kg/m^2^) was calculated from height and weight reported at baseline, studied both as a continuous variable, and categorized according to the World Health Organization’s guidelines: underweight (<18.5), normal weight (18.5-24.9), overweight (25.0-29.9), and obese (≥30.0).^40^ Based on self-reported records of consumption by age, smoking status was defined as never, former, or current smokers below (<) or above (≥) average daily consumption (12 cigarettes). For the analyses, birth cohort was defined as 1915-1924, 1925-1934, …, 1955-1964, 1965-1979.

### Healthy worker survivor bias and directed acyclic graphs

Since 1990, an offshore worker’s medical certificate must be renewed every second year.^41^ Potentially invalidating health issues include overweight, cardiovascular disease, elevated blood pressure, high cholesterol values, and diabetes mellitus,^42^ conditions known or suspected to increase kidney cancer risk, and often associated with changes in work status: reduced workload, switching jobs, etc. Termination or change of work may lead to lower occupational exposure in workers at higher risk of kidney cancer, biasing or even inverting an exposure-outcome association.^43^^,^^44^

**Figure 1:**
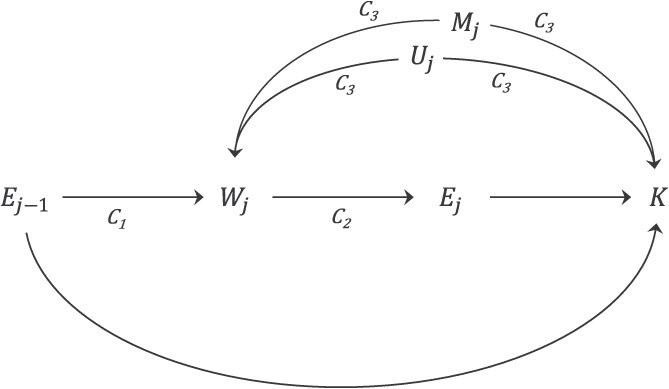
Directed acyclic graph illustrating healthy worker survivor bias: *j* is age, *E* is exposure offshore (single agent or exposure condition), *W* is work status, *M* is measured and *U* is unmeasured common causes of *W* and *K*, and *K* is kidney cancer diagnosis. Time-varying confounding by work status is depicted by the component associations (pathways) *C*_1_, *C*_2_, and *C*_3_ (adapted from Naimi et al. 2013^45^; and Bertke et al. 2021^46^).

An association between work status and kidney cancer, confounded by the conditions described above, can be a component of HWSB, corresponding to the pathway or “component association” *C*_3_ in the directed acyclic graph (DAG) in Figure 1.^45^^,^^46^ DAGs are commonly used to illustrate structures of HWSB with time-varying exposure and confounding,^20^ and we adopt the terminology of Naimi et al.^45^ when referring to component associations of HWSB. Analysis of such associations underlying HWSB has also been referred to as “path analysis”^47^ (which is distinct from other formal definitions of path analysis).^48^ In Figure 1, the subscript *j* is age, *E_j_* is the exposure at age *j*, and *E*_*j–*1_ is prior exposure. *W_j_* is the work status (employed or not employed offshore at age *j*, or total employment duration at age *j* as a function of work status), *K* is the outcome (kidney cancer), and *M_j_* and *U_j_* are measured and unmeasured variables, respectively, and common causes of *W_j_* and *K*. The DAG also includes 2 other component associations, *C*_1_ and *C*_2_. *C*_1_ is the association between prior exposure (*E*_*j–*1_) and work status (*W_j_*), and *C*_2_ the association between work status (*W_j_*) and exposure (*E_j_*), while *C_3_*, as already noted, is the association between work status (*W_j_*) and kidney cancer (*K*). The component association *C*_2_ is inherently present in our data as termination of offshore employment would lead to no further occupational exposure. Please see the next section and supplementary file for technical description including pathways evaluated in the assessment of component associations *C*_1_ and *C*_3_.

### Statistical analysis

Weighted Cox regression, stratified by birth cohort, with age as the timescale and standard errors derived from robust variance, was used to estimate HRs and 95% CIs for associations between occupational exposures and kidney cancer. Stratification by birth cohort was used because kidney cancer incidence rate in males increased about 30% in Norway during follow-up.^30^ Entry time was age at start of follow-up, and exit was age at diagnosis of kidney cancer, emigration, death, or end of follow-up, whichever occurred first. Kidney cancer cases were assigned weights = 1 and noncases in the subcohort weights according to their inverse sampling fractions in 5-year birth cohort strata.^34^ Tests for trend were performed using the median value within each exposure category.

Exposure-response relationships were displayed in graphs using restricted cubic splines (RCS) with 3 knots corresponding to the 0.10, 0.50, and 0.90 quantile distribution of exposed workers. Exposure lagging was performed by disregarding exposure during the last 10 or 20 years prior to any year of observation during follow-up. This would account for cancer latency and for unobserved exposure occurring after baseline.

In assessing component associations *C*_1_ and *C*_3_, we used Cox regression also weighted by the inverse sampling fractions, and with age as the time scale. For *C*_1_, we analyzed the association between employment termination 1968-1996 and prior exposure, defined as cumulative exposure during a 3-year period prior to observation. For *C*_3_, we assessed the association between total employment duration (at baseline) and kidney cancer during follow-up, and we additionally adjusted for smoking and BMI, and stratified it by birth cohort. In sensitivity analyses for *C*_3_, we further adjusted for individual prior exposure. Detailed descriptions of these analyses are given in the Supplementary material.

Upon estimating associations between occupational exposures and kidney cancer, our analytical models were built on postulated associations and potential confounding illustrated by the DAG in Figure 1. In model 1, we adjusted for age only (as the time scale). In model 2, we additionally adjusted for BMI (continuous) and smoking history at baseline, representing our measured variables (*M*) associated with work status and kidney cancer, and acting as a part of component association *C*_3_. Model 3 was additionally adjusted for total (cumulative) employment duration (at baseline, continuous), as a measure of work status (*W*) to address unmeasured time-varying confounding (*U*), also acting through component association *C*_3_*.* If component association *C*_1_ is active, adjustment for total employment duration may introduce collider stratification bias, and g-methods could be considered. We were not able to apply g-methods,^49^^,^^50^ as we had no information on work status during follow-up. We rationalize the chosen approach by noting that not all exposures may impact work status, which would make adjustment by total employment duration appropriate to control this bias. Further, in cases where exposures may impact work status, we note that the induced collider stratification bias is often expected to be smaller than the confounding bias that would otherwise be present.^51^ Thus, because the lack of work history data after baseline precluded use of g-methods, we opted for incomplete adjustment in preference to no adjustment.

The proportional hazard assumption was evaluated using Schoenfeld residuals for each variable and found satisfactory. To facilitate comparison with earlier studies of the role of BMI and smoking, we estimated HRs for the associations with kidney cancer in separate models.

To reduce the impact on kidney cancer risk from diagnostic procedures and cancer therapy during follow-up, and to analyze a more uniform subtype of kidney cancer, we performed a sensitivity analysis of cumulative exposure, restricting the outcome to first primary renal cell carcinoma (RCC), by censoring subjects on the date of any first primary cancer other than RCC, and disregarding RCC diagnosed at the same date as one or more other cancers (tied cancers) (Figure S2).

We had up to 5% missing information in the covariates smoking and BMI. For the multivariable model, multiple imputation with chained equations was used to provide 20 complete datasets.^52^^,^^53^ All statistical analyses were conducted with the statistical software Stata (version 17).^54^ The significance level was 5%, and all tests were 2-sided.

## Results

Median age at kidney cancer diagnosis was 63 years (range 38-88, Table 1). Positive associations between kidney cancer and BMI or smoking were confirmed in supplementary analyses (Tables S2 and S3).

**Table 1:** Characteristics (1998) of the study sample (*n* = 2259) in the Norwegian offshore petroleum workers cohort.

**Variables**	**Cases (*n* = 169)**		**Noncases (*n* = 2090)**	
Age at baseline, years, median (range)	49	(23-70)	54	(20-80)
Age at diagnosis, years, median (range)	63	(38-88)	NA	NA
Birth cohort, *n* (%)				
1915-1924	0	(0.0)	25	(1.2)
1925-1934	6	(3.6)	336	(16.1)
1935-1944	44	(26.0)	761	(36.4)
1945-1954	72	(42.6)	686	(32.8)
1955-1964	38	(22.5)	236	(11.3)
1965-1979	9	(5.3)	46	(2.2)
BMI (kg/m^2^), median (range)	26.5	(20.0-54.1)	25.6	(13.7-60.8)
BMI, kg/m^2^, *n* (%)				
12-18.4	0	(0.0)	6	(0.3)
18.5-24.9	54	(32.0)	841	(40.2)
25.0-29.9	83	(49.1)	1035	(49.5)
≥30.0	27	(16.0)	175	(8.4)
Missing	5	(3.0)	33	(1.6)
Smoking status, *n* (%)				
Never	40	(23.7)	450	(21.5)
Former	57	(33.7)	857	(41.0)
Current <12 cigarettes per day	33	(19.5)	355	(17.0)
Current ≥12 cigarettes per day	32	(18.9)	357	(17.1)
Missing	7	(4.1)	71	(3.4)
Work history				
Year of first employment, *n* (%)				
1965-1974	14	(8.3)	300	(14.6)
1975-1984	92	(54.4)	1244	(59.5)
1985-1998	63	(37.3)	546	(26.1)
Total employment duration, years, median (range)^a^	11.0	(0.3-27.5)	12.4	(0.1-33.5)
Total employment duration, years by quartiles, *n* (%)^a^				
>0 to <6.0	54	(32.0)	524	(25.1)
6.0 to <12.2	47	(27.8)	505	(24.2)
12.2 to <18.3	39	(23.1)	531	(25.4)
18.3-33.5	29	(17.2)	530	(25.4)

Abbreviations: BMI, body mass index; NA, not applicable.

a Complete work history, that is, up to 8 employments as an offshore worker before baseline.

**Figure 2:**
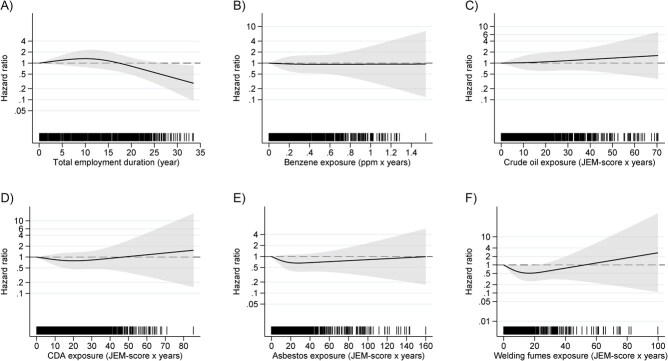
Exposure-risk curves with 95% confidence intervals (shaded area) for nonlinear models: using restricted cubic splines with 3 knots corresponding to the 0.10, 0.50, and 0.90 quantiles among exposed workers in each respective plot. (A) Total employment duration, using model 2 (adjusted for age as time scale, body mass index, and smoking), and knot placement at 1.8, 12.1, and 21.5 years. (B-F) Cumulative exposures applying 20-year lag and model 3 (model 2 + total employment duration, as a spline), for (B) benzene with knot placement at 0.01, 0.12, and 0.63 ppm × years; (C) crude oil skin exposure, knot placement at 1.7, 12.0, and 30.5 JEM-score × years; (D) CDA, knot placement at 2.0, 16.0, and 38.0 JEM-score × years; (E) asbestos, knot placement at 1.4, 11.8, and 37.4 JEM-score × years; and (F) welding fumes, knot placement at 0.5, 2.7, 7.2, 16.0, and 40.0 JEM-score × years. Rugs on the x-axis illustrate the distribution of individual data points representing each worker’s maximum exposure during follow-up. Abbreviations: CDA, chlorinated degreasing agent; JEM, job-exposure matrices; ppm, parts per million.

An inverse association was found between kidney cancer and total employment duration, which reflects work status prior to follow-up (component association *C*_3_) (model 2, Table 2). We found a reduced kidney cancer HR = 0.80 (95% CI, 0.64-1.00) per decade increase in total employment duration (*P* = .05) (model 2, Table 2), which remained largely unchanged with additional adjustment for the individual exposures under study (Table S4). The RCS curve for this association declined from reference after about 18 years of total employment duration (model 2, Figure 2A).

**Table 2:** Hazard ratios of kidney cancer and 95% CI according to total employment duration, cumulative exposure, and duration in the work category surface treatment in the Norwegian offshore petroleum workers cohort followed from 1999 to 2021.

**Variables**	**Cases**	**Noncases**	**Model 1** ^**a**^		**Model 2** ^**b**^		**Model 3** ^**c**^	
			**HR**	**95% CI**	**HR**	**95% CI**	**HR**	**95% CI**
Total employment duration, years								
>0 to <6.0	54	524	1.00	(Reference)	1.00	(Reference)		
6.0 to <12.2	47	505	1.00	0.66-1.53	1.02	0.67-1.55		
12.2 to <18.3	39	531	0.83	0.53-1.30	0.84	0.54-1.32		
18.3 to <33.5	29	530	0.62	0.38-1.01	0.60	0.37-0.99		
*P*-trend^d^			*.047*		*.042*			
Continuous 10-unit	169	2090	0.80	0.65-1.00	0.80	0.64-1.00		
*P* value			*.046*		*.046*			
Cumulative exposures								
Benzene (ppm × years)								
0	54	640	1.00	(Reference)	1.00	(Reference)	1.00	(Reference)
>0.000 to <0.059	49	473	1.09	0.72-1.65	1.11	0.73-1.69	1.07	0.70-1.64
0.059 to <0.219	36	486	0.92	0.59-1.44	0.96	0.61-1.50	0.99	0.63-1.56
0.219 to <1.542	30	491	0.78	0.48-1.25	0.76	0.47-1.23	0.85	0.52-1.41
*P*-trend^d^			*.200*		*.159*		*.434*	
Crude oil (JEM-score × years)								
0	38	505	1.00	(Reference)	1.00	(Reference)	1.00	(Reference)
>0 to <7.4	47	525	0.89	0.56-1.41	0.91	0.57-1.45	0.80	0.49-1.29
7.4 to <17.6	50	522	0.97	0.62-1.51	0.98	0.63-1.54	1.06	0.68-1.67
17.6-70.5	34	538	0.71	0.44-1.16	0.73	0.45-1.19	0.94	0.54-1.62
*P*-trend^d^			*.211*		*.254*		*.958*	
CDA (JEM-score × years)								
0	26	334	1.00	(Reference)	1.00	(Reference)	1.00	(Reference)
>0 to <9.2	58	575	1.00	0.61-1.67	1.00	0.60-1.67	0.87	0.50-1.53
9.2 to <23.0	44	589	0.84	0.50-1.40	0.86	0.51-1.44	0.90	0.54-1.50
23.0-85.4	41	592	0.81	0.48-1.36	0.79	0.46-1.34	0.97	0.56-1.71
*P*-trend^d^			*.277*		*.246*		*.952*	
Asbestos (JEM-score × years)								
0	40	457	1.00	(Reference)	1.00	(Reference)	1.00	(Reference)
>0 to <7.1	56	533	0.97	0.63-1.49	0.99	0.64-1.53	0.95	0.60-1.49
7.1 to <16.7	42	544	0.79	0.50-1.25	0.81	0.51-1.28	0.83	0.53-1.31
16.7-160.0	31	556	0.63	0.39-1.04	0.62	0.38-1.03	0.69	0.40-1.18
*P*-trend^d^			*.037*		*.032*		*.157*	
Welding fumes (JEM-score × years)								
0	108	1157	1.00	(Reference)	1.00	(Reference)	1.00	(Reference)
>0 to <3.8	28	304	0.89	0.57-1.39	0.93	0.59-1.46	0.89	0.56-1.39
3.8 to <12.5	19	312	0.71	0.42-1.18	0.67	0.39-1.14	0.69	0.40-1.18
12.5-100.5	14	317	0.53	0.30-0.94	0.53	0.30-0.95	0.59	0.32-1.07
*P*-trend^d^			*.023*		*.022*		*.059*	
Surface treatment								
Never	160	2031	1.00	(Reference)	1.00	(Reference)	1.00	(Reference)
Ever	9	59	2.13	1.01-4.52	2.22	1.04-4.72	2.21	1.04-4.69
Duration, years								
0	160	2031	1.00	(Reference)	1.00	(Reference)	1.00	(Reference)
>0 to <8.1	6	28	2.92	1.15-7.43	3.09	1.22-7.84	2.87	1.13-7.26
8.1 to <21.3	3	31	1.38	0.40-4.81	1.42	0.41-4.98	1.52	0.44-5.29
*P*-trend^d^			*.295*		*.272*		*.222*	

Abbreviations: CDA, chlorinated degreasing agents; JEM, job-exposure matrices; ppm, parts per million.

a Model 1: Cox regression adapted to the case-cohort design, stratified by birth cohort and adjusted for age (as time scale).

b Model 2: model 1 + body mass index and smoking. Missing values in BMI (*n* = 38) and smoking (*n* = 78) were imputed.

c Model 3: model 2 + total employment duration.

d Modeled by using the medians within each category to test for linear trend.

There were associations between prior exposure (*E*_*j–*1_) and subsequently leaving work (ie, work status *W_j_*, component association *C*_1_) that differed depending on the agent (Table S5). For benzene and crude oil, workers with a high cumulative exposure during the last 3 years were less likely to leave work (*P*-trend < .001). For asbestos, those with low and medium exposure levels were less likely to leave work, while workers with high exposure levels were suggestively more likely to leave (HR = 1.20; 95% CI, 0.96-1.50). For exposure to CDA or welding fumes, no association was suggested (Table S5). Table 3 presents an overview of the active HWSB components by agent.

**Table 3:** Overview of active HWSB components (pathways) illustrated in the DAG by agent in this study, and the analytical strategies to reduce bias upon estimating the association between occupational exposure and kidney cancer.

	**Component Associations of HWSB**			**Adjusting for HWSB**	
**Agent**	***C***_**1**_ **Prior exposure (*E***_***j−*1**_**) and work status (*W***_***j***_**)**	***C***_**2**_ **Work status (*W***_***j***_**) and exposure (*E***_***j***_**)**	***C***_**3**_ **Work status (*W***_**j**_**) and kidney cancer (K)**	**Analysis method used in this study** ^**a**^	**Analysis method recommended**
Benzene	Present	Present	Present	Regression adjustment	G-methods
Crude oil	Present	Present	Present	Regression adjustment	G-methods
CDA	Absent	Present	Present	Regression adjustment	Regression adjustment
Asbestos	Present	Present	Present	Regression adjustment	G-methods
Welding fumes	Absent	Present	Present	Regression adjustment	Regression adjustment

Abbreviations: CDA, chlorinated degreasing agents; DAG, directed acyclic graph; HWSB, healthy worker survivor bias; *j*, age.

a Cox regression adapted to the case-cohort design, stratified by birth cohort and adjusted for age (as time scale), total employment duration, body mass index, and smoking (model 3).

The analyses of kidney cancer risk and occupational exposure to all agents separately with models 1-2 suggested a reduction in the HR with increasing cumulative exposure, particularly for asbestos and welding fumes, with *P-*trends of .03 and .02 (Table 2). With additional adjustment for total employment duration (model 3), no associations were found, except remaining inverse trends for asbestos and welding fumes (*P*-trend = .16 and *P*-trend = .06, respectively; model 3, Table 2). For average intensity of exposure, no association was seen for any of the agents (models 1-3, Table S6).

Workers ever employed in surface treatment offshore had increased risk of kidney cancer (HR = 2.21; 95% CI, 1.04-4.69; 9 exposed cases) compared to workers never reporting such work, although no trend was observed with increasing duration (model 3, Table 2).

With a 20-year lag of exposures, an increase was suggested in the HRs for all tertiles of cumulative exposure to benzene, crude oil, and CDA both for models 2 and 3. The highest HRs in the upper exposure tertiles were seen in model 3 (20-year exposure lag and adjusted for total employment duration); still there were no trends (.30 ≤ *P*-trend ≤ .99) (Table 4). For asbestos and welding fumes, lagging of exposures did not induce much change in the inverse trends, and most of the HRs remained below unity (Table 4). The RCS curves for benzene, crude oil, and CDA based on model 3 and 20-year lag of exposure showed no relationship between exposure and outcome (Figure 2B-D). For asbestos and welding fumes, the RCS curve was mainly below unity (Figure 2E and F).

**Table 4:** Hazard ratios of kidney cancer and 95% CI according to cumulative exposure with 10- or 20-year exposure lag in the Norwegian offshore petroleum workers cohort followed from 1999 to 2021.

**Cumulative exposure**	**Cases**	**Person-years**	**Model 2** ^**a**^ **and 10-year lag**		**Model 3** ^**b**^ **and 10-year lag**		**Cases**	**Person-Year**	**Model 2** ^**a**^ **and 20-year lag**		**Model 3** ^**b**^ **and 20-year lag**	
			**HR**	**95% CI**	**HR**	**95% CI**			**HR**	**95% CI**	**HR**	**95% CI**
Benzene (ppm × years)												
0	55	13 713.2	1.00	(Reference)	1.00	(Reference)	60	17 914.8	1.00	(Reference)	1.00	(Reference)
>0.000 to <0.059	48	10 276.3	1.07	0.71-1.63	1.04	0.68-1.59	52	10 646.2	1.26	0.85-1.87	1.26	0.84-1.88
0.059 to <0.219	38	9776.2	1.00	0.65-1.56	1.05	0.67-1.63	33	8670.0	1.03	0.66-1.62	1.14	0.72-1.81
0.219 to <1.542	28	9163.5	0.74	0.45-1.21	0.83	0.50-1.39	24	5698.2	0.98	0.57-1.68	1.18	0.67-2.08
*P*-trend^c^			*.155*		*.427*				*.650*		*.768*	
Crude oil (JEM-score × years)												
0	39	10 640.7	1.00	(Reference)	1.00	(Reference)	48	15 562.3	1.00	(Reference)	1.00	(Reference)
>0 to <7.4	48	11 623.4	0.91	0.57-1.43	0.80	0.50-1.28	54	13 456.3	1.00	0.67-1.51	0.98	0.65-1.48
7.4 to <17.6	51	11 611.1	0.96	0.62-1.50	1.07	0.69-1.66	48	9475.8	1.12	0.72-1.73	1.39	0.87-2.22
17.6-70.5	31	9054.1	0.74	0.45-1.22	0.97	0.55-1.70	19	4434.8	0.85	0.47-1.54	1.23	0.64-2.36
*P*-trend^c^			*.301*		*.863*				*.776*		*.301*	
CDA (JEM-score × years)												
0	27	7408.7	1.00	(Reference)	1.00	(Reference)	38	12 597	1.00	(Reference)	1.00	(Reference)
>0 to <9.2	58	12 520.0	1.00	0.61-1.66	0.89	0.52-1.52	64	14 036.7	1.17	0.75-1.81	1.15	0.73-1.79
9.2 to <23.0	45	12 151.7	0.87	0.52-1.45	0.93	0.56-1.55	44	10 445.8	1.00	0.62-1.63	1.14	0.68-1.90
23.0-85.4	39	10 848.8	0.81	0.48-1.38	1.03	0.58-1.82	23	5849.7	0.80	0.45-1.44	1.02	0.54-1.92
*P*-trend^c^			*.307*		*.813*				*.263*		*.986*	
Asbestos (JEM-score × years)												
0	41	9877.7	1.00	(Reference)	1.00	(Reference)	50	14 573.9	1.00	(Reference)	1.00	(Reference)
>0 to <7.1	55	11 927.6	0.95	0.62-1.47	0.91	0.58-1.41	64	12 593.7	1.16	0.78-1.73	1.15	0.77-1.72
7.1 to <16.7	43	11 112.2	0.83	0.53-1.30	0.87	0.55-1.36	32	8803.6	0.76	0.48-1.22	0.82	0.50-1.32
16.7-160.0	30	10 011.8	0.64	0.39-1.06	0.73	0.42-1.26	23	6958.1	0.72	0.42-1.23	0.81	0.46-1.43
*P*-trend^c^			*.058*		*.273*				*.080*		*.302*	
Welding fumes (JEM-score × years)												
0	109	24 459.7	1.00	(Reference)	1.00	(Reference)	114	27 562.4	1.00	(Reference)	1.00	(Reference)
>0 to <3.8	27	6472.1	0.89	0.57-1.40	0.85	0.54-1.33	26	6349.8	0.90	0.57-1.41	0.87	0.55-1.37
3.8 to <12.5	19	6338.5	0.66	0.39-1.13	0.69	0.40-1.18	20	5614.1	0.77	0.46-1.30	0.82	0.49-1.39
12.5-100.5	14	5658.9	0.56	0.31-1.00	0.62	0.34-1.13	9	3402.9	0.52	0.26-1.07	0.59	0.29-1.22
*P*-trend^c^			*.036*		*.092*				*.057*		*.136*	
**Surface treatment**	**Cases**	**Person-years**	**Model 2** ^**a**^ **and 10-year lag**		**Model 3** ^**b**^ **and 10-year lag**		**Cases**	**Person-years**	**Model 2** ^**a**^ **and 20-year lag**		**Model 3** ^**b**^ **and 20-year lag**	
			**HR**	**95% CI**	**HR**	**95% CI**			**HR**	**95% CI**	**HR**	**95% CI**
Never	160	41 795.3	1.00	(Reference)	1.00	(Reference)	162	42 065.1	1.00	(Reference)	1.00	(Reference)
Ever	9	1133.9	2.25	1.05-4.79	2.24	1.06-4.75	7	864.2	1.98	0.85-4.62	1.99	0.86-4.60
Duration, years												
0	160	41 795.3	1.00	(Reference)	1.00	(Reference)	162	42 065.1	1.00	(Reference)	1.00	(Reference)
>0 to <8.1	6	640.7	2.95	1.18-7.38	2.75	1.10-6.88	6	638.1	2.57	1.05-6.28	2.48	1.01-6.05
8.1 to <21.3	3	493.2	1.52	0.43-5.37	1.64	0.47-5.73	1	226.1	0.82	0.10-6.61	0.90	0.11-7.22
*P*-trend^c^			*.246*		*.196*				*.577*		*.498*	

Abbreviations: CDA, chlorinated degreasing agents; JEM, job-exposure matrices; ppm, parts per million.

a Model 2: Cox regression adapted to the case-cohort design, stratified by birth cohort and adjusted for age (as time scale), body mass index, and smoking. Missing values in BMI (*n* = 38) and smoking (*n* = 78) were imputed.

b Model 3: model 2 + total employment duration.

c Modeled by using the medians within each category to test for linear trend.

In a sensitivity analysis, we restricted the outcome to first primary RCC, excluding 39 non–first-primary kidney cancers and 7 non-RCC (Figure S2). No clear trend emerged in unlagged analyses of cumulative exposure, except for a suggested inverse trend for asbestos (model 3, Table S7). Workers employed in surface treatment offshore had increased HR = 3.32 (95% CI, 1.53 to 7.19; 9 exposed cases) compared to the unexposed (model 3, Table S7). With a 20-year exposure lag applied to model 3, the highest HR estimates for cumulative exposure to benzene and crude oil appeared in the highest exposure tertile with HR = 1.90 (95% CI, 0.98-3.68) for benzene and HR = 1.44 (95% CI, 0.64-3.28) for crude oil, but still with no trend (Table S8). For CDA, the highest exposure tertile retained the lowest HR = 0.69 (95% CI, 0.30-1.56).

## Discussion

In our analyses of kidney cancer risk among NOPWs, we found a suggested HWSB in the naïve models (models 1 and 2), supported by component associations between work status, exposures, and outcome. Some of this downward bias was alleviated for benzene, crude oil, and CDA, with HR estimates close to unity, when we adjusted for total employment duration (model 3). An additional increase in the HRs with model 3 was suggested in the upper exposure categories when we applied a 20-year exposure lag, reinforced when the outcome was restricted to first primary RCC, although not for CDA, and with no exposure-related trends. Additionally, a small occupational group engaged in surface treatment had increased HR for kidney cancer, based on only 9 exposed cases. For asbestos and welding fumes, the inverse trends did not change much with adjustment for total employment and lagging of exposures, and most of the HRs remained below unity (Table 3).

Analyses were adjusted for BMI and smoking, but we lacked information on other kidney cancer risk factors such as diabetes and hypertension. Such conditions are likely to invalidate health certificates and thus preclude individuals from further work offshore, to cause sick leave, or lead to health-related retirement.^41^ This situation can add to the HWSB in our data, and it prompted us to address this time-varying confounding by adjusting for total employment duration. Sensitivity analyses excluding older birth cohorts and/or those actively employed by 1998 did not suggest that the inverse exposure-kidney cancer trend was linked to differential assessment of exposure (data not shown).

Healthy worker survivor bias can be more complicated to handle when termination of work is caused by factors in the work environment, particularly by the exposures under study. Such a bias can be difficult to adjust for by traditional methods, as collider stratification bias may affect the estimates.^20^^,^^55^ It can then be necessary to use more advanced methods such as g-methods, which we were unable to use due to the lack of work history data during follow-up. Indeed, there was some suggestion that g-methods would have given less biased risk estimates. Asbestos, which was in legal use until 1985 in Norway, was the only exposure suggestively associated with a higher risk of leaving work after high exposure. In contrast, workers were less likely to leave work with increasing exposure to benzene or crude oil. Standard views of HWSB hold that leaving work would be more probable at higher exposures. We note that such an inverse finding has also been observed with asbestos^45^ and silica,^56^ while Bertke et al.^46^ noted the direction of this association depended on whether exposure was characterized as cumulative exposure or average exposure. Regardless of the direction of this association (more or less likely to leave work), adjusting for total employment duration could introduce collider stratification bias, which must be weighed against bias reduction from controlling confounding by work status.

The association between kidney cancer and average intensity of exposure, an exposure metric that is less dependent on exposure duration, seemed to be less influenced by HWSB, as was expected (Table S6).

In our study, the risk of kidney cancer increased 34% per 5 increments in BMI at baseline, which is similar to findings reported in other studies, within and outside the petroleum industry.^57-59^ On the contrary, for the association between work status and kidney cancer, it seemed that BMI and smoking were no strong confounders, at least not alone, as no appreciable change was found when they were adjusted for (Table 2). We had no information to adjust for lifetime variation of these variables.

Other conditions that may obscure an exposure-kidney cancer association in our NOPW cohort include left truncation in the cohort establishment. Cohort membership was based on the 1998 survey, and in this respect, the cohort is a selected group, as survey participants had to be alive, and responders probably were healthier than the nonrespondents.^60^ Further, we decided to exclude from follow-up individuals with prevalent cancers at baseline to avoid information bias by differential reporting. We did not specifically assess TCE but rather a broader group of chlorinated solvents, and exposure misclassification could have resulted in the null finding for the agent CDA.^61^

Increased risks of kidney cancer (incident cancers or deaths) compared to the general population have been reported among workers exposed to petroleum products in many countries, such as the United States, Canada, UK, Germany, and the Nordic countries.^6^^,^^9^^,^^11^^,^^13^^,^^14^^,^^62^^,^^63^ Also, suspected causal agents have been reviewed by IARC several times, with no firm conclusions.^7^^,^^28^^,^^64^ For the period 1999-2022, our NOPW cohort showed a kidney cancer standardized incidence ratio (SIR) of 0.99 (95% CI, 0.85-1.14) compared with the general population (unpublished data). An earlier study of a registry-based cohort of Norwegian offshore workers, compared with occupationally active onshore workers, found an overall kidney cancer SIR of 1.3 (95% CI, 0.8-1.9) for the period 1981-2003.^65^ Such a registry-based study may have been less biased from healthy hire or left truncation, compared to our survey-based NOPW cohort.

The present study demonstrates that negative findings of kidney cancer risk among petroleum workers may be due to selection issues related to ill health, which could mask a weak or moderate exposure response.

Strengths of our study include a relatively large number of kidney cancers and a nationwide cancer registry providing virtually complete and high-quality incidence data. Information on work histories, lifestyle, and anthropometric factors was collected at baseline, enabling a prospective follow-up of cancer. Industry-specific exposures were assessed by experts in the field.

A major limitation is the lack of exposure and work history data during follow-up. This shortcoming precluded the implementation of g-methods as an approach to control for HWSB. Misclassification of exposure is often present, but the organizing of JEMs at the group level leads to a Berkson-type error, which does not substantially bias the estimates of relative risk.^66^

In conclusion, we cannot exclude the possibility that occupational exposures to petroleum-derived hydrocarbons may contribute to kidney cancer development. Depending on the agent, there were indications that more advanced statistical methods could be useful in avoiding residual time-varying confounding.

## Supplementary Material

Web_Material_kwaf039

## Data Availability

Data may be obtained from a third party and are not publicly available. The data that support the findings of this study are available from the CRN (cohort data and cancer data) and the National Population Register (death and emigration data), but restrictions apply to the availability of these data, which were used under license for the current study, and so are not publicly available. Requests for data sharing/case pooling for projects with necessary approvals and legal basis according to the EU General Data Protection Regulation (GDPR) may be directed to principal investigator Dr. J.S.S. (email: jo.stenehjem@kreftregisteret.no).
