# Supplementary material for: Occupational exposures and kidney cancer among 25 000 male offshore petroleum industry workers: relative risks and healthy worker survivor bias
Source: Am J Epidemiol. 2025 Feb 28;195(1):81–91. doi: 10.1093/aje/kwaf039 (PMC12780764; doi:10.1093/aje/kwaf039)
Supplement: Web_Material_kwaf039 [file web_material_kwaf039.zip › Supplementary-Material_CLEAN_AJE-00350-2024_R2.docx]

**Supplementary Material**

**Title:** Occupational exposures and kidney cancer among 25 000 male offshore petroleum industry workers: relative risks and healthy worker survivor bias

**Authors:** Nita K Shala, Marit B Veierød, Ronnie Babigumira, Leon AM Berge, Sven O Samuelsen, Jorunn Kirkeleit, Magne Bråtveit, Melissa C Friesen, Alexander P Keil, Debra T Silverman, Nathaniel Rothman, Lan Qing, Jo S Stenehjem, Tom K Grimsrud.

**Content**

- **Table S1**: Number of kidney cancer cases by histological subtype
- **Figure S1**: Overview of study design, exclusions and study sample: All kidney cancers
- **Figure S2**: Overview of study design, exclusions and study sample: First primary renal cell carcinoma
- **Table S2**: Associations between body mass index and kidney cancer
- **Table S3**: Associations between smoking and kidney cancer
- **Technical description** explaining the assessment of component association *C_3_*
  - **Table S4**: Assessing the association between total employment duration and kidney cancer
- **Technical description** explaining the assessment of component association *C_1_*
  - **Table S5**: Assessing the association between prior exposure and subsequently leaving work
- **Table S6**: Average intensity of exposure and kidney cancer
- **Table S7**: Total employment duration and cumulative exposures offshore and first primary renal cell carcinoma, un-lagged exposure
- **Table S8**: Cumulative exposures offshore and first primary renal cell carcinoma, exposure with 10-yr and 20-yr lags

| **Table S1.** Numbers of kidney cancer cases by histological subtype from the study sample among males in the Norwegian Offshore Petroleum Workers (NOPW) cohort followed 1999–2021 | | | | |
| --- | --- | --- | --- | --- |
| **Cancer type** | **Number of cases** | | | |
| Kidney (C64: all cases) | 169 |  |  |  |
| Renal cell carcinoma (RCC) |  | 159 |  |  |
| Adenocarcinoma RCC^*^ |  |  | 146 |  |
| Clear cell^1 *^ |  |  |  | 109 |
| Papillary^2 *^ |  |  |  | 24 |
| NOS^3 *^ |  |  |  | 13 |
| Chromophobe RCC^4 *^ |  |  | 7 |  |
| Sarcomatoid RCC^5 *^ |  |  | 4 |  |
| Cyst-associated RCC^6 *^ |  |  | 2 |  |
| Other^7 *^ |  | 6 |  |  |
| Unknown^8 *^ |  | 4 |  |  |
| ^1-8^ Codes from the International Classification of Diseases Oncology 3^rd^ revision (ICD‑O‑3) used to classify histological subtypes of kidney cancer: | | | | |
| ^1^ 8310 | | | | |
| ^2^ 8260 | | | | |
| ^3^ 8312 | | | | |
| ^4^ 8317 | | | | |
| ^5^ 8318 | | | | |
| ^6^ 8316 | | | | |
| ^7^ 8010, 8140, 8323, 8890 | | | | |
| ^8^ 8000 | | | | |
| ^*^ Not analysed separately | | | | |


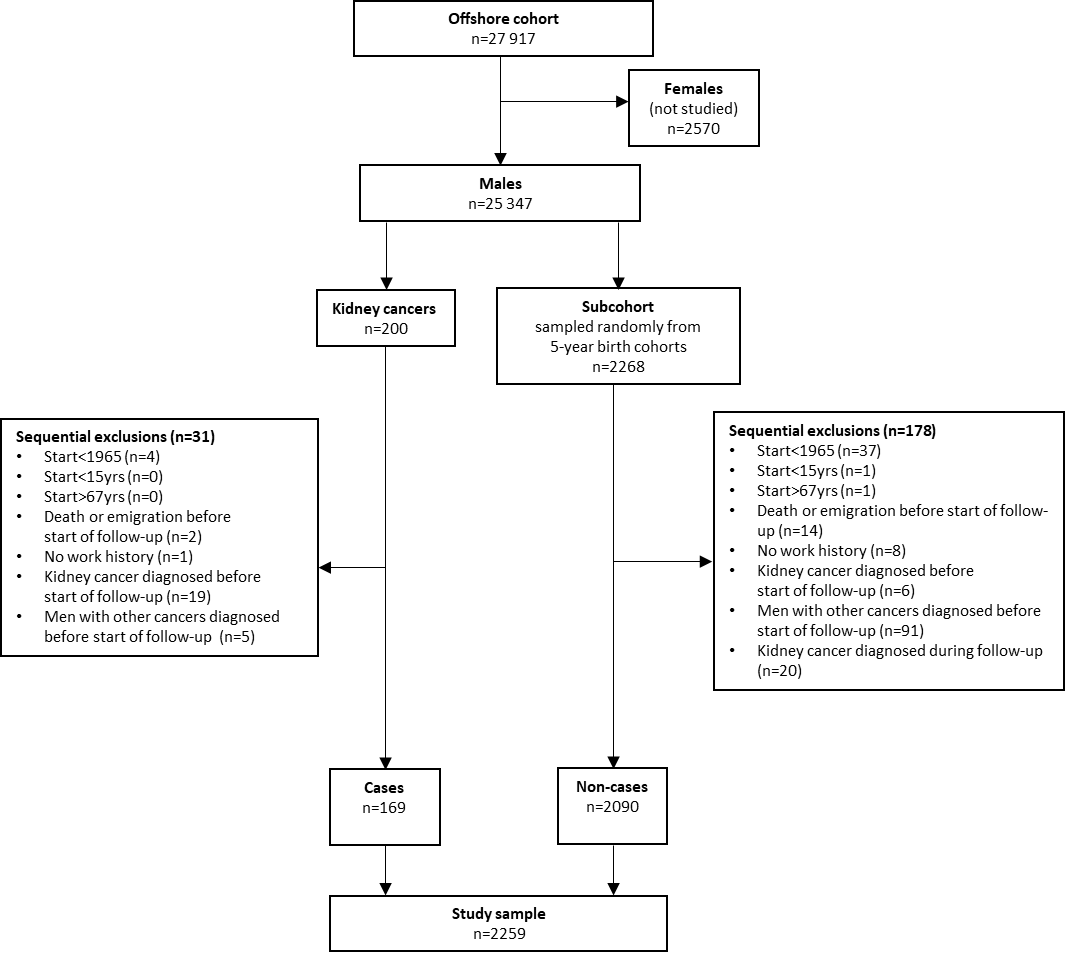


**Figure S1.** Overview of study design and study sample (cases including all kidney cancers)


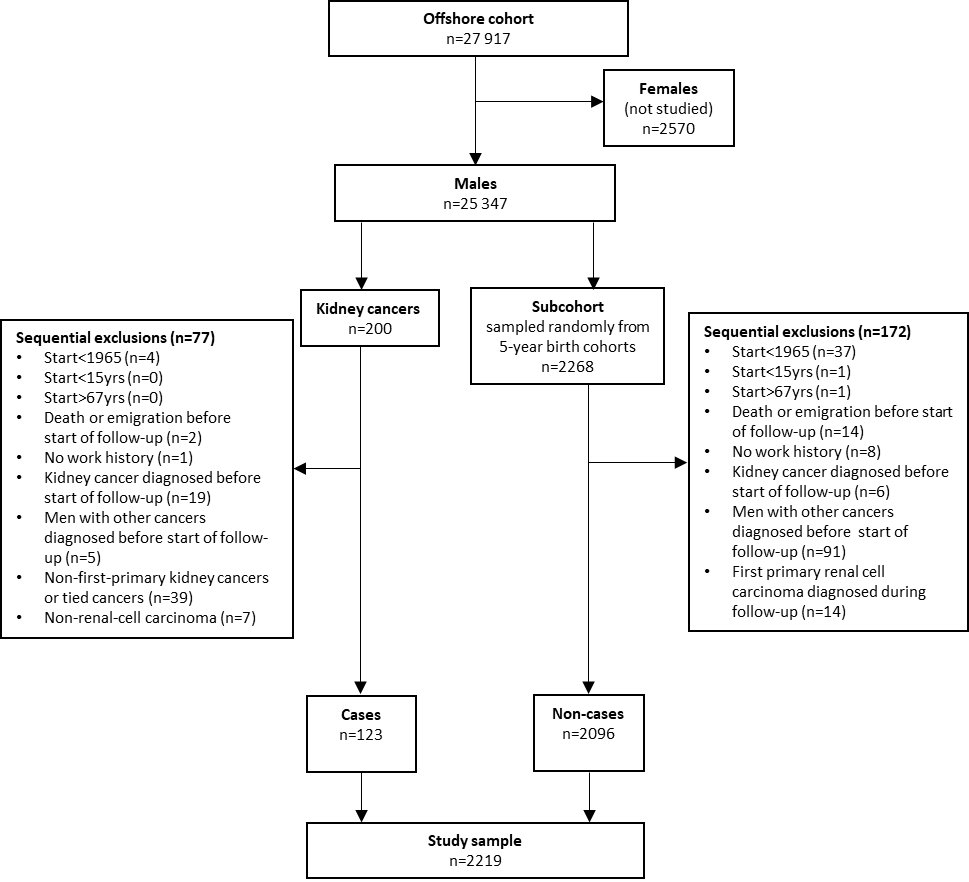


**Figure S2.** Overview of study design and study sample (cases including first primary renal cell carcinoma (RCC), only)

| **Table S2.** Hazard ratios (HR) of kidney cancer and 95% confidence intervals (95% CI) according to body mass index (BMI) at baseline in the Norwegian Offshore Petroleum Workers cohort followed 1999–2021 | | | | | | | | | | |
| --- | --- | --- | --- | --- | --- | --- | --- | --- | --- | --- |
| **BMI (kg/m^2^)** | **Cases** | **Non-cases** | **Model 1**^a^ | | | | **Model 2**^b^ | | | |
|  |  |  | **HR** | **95% CI** | | | **HR** | **95% CI** | | |
| 12–24.9 ^c^ | 54 | 847 | 1.00 | (reference) | | | 1.00 | (reference) | | |
| 25.0–29.9 | 83 | 1 035 | 1.26 | 0.88 | – | 1.82 | 1.29 | 0.89 | – | 1.85 |
| ≥30.0 | 27 | 175 | 2.36 | 1.42 | – | 3.91 | 2.44 | 1.46 | – | 4.07 |
|  |  |  |  |  |  |  |  |  |  |  |
| Continuous 1-unit | 169 | 2090 | 1.06 | 1.02 | – | 1.10 | 1.06 | 1.02 | – | 1.10 |
| Continuous 5-unit | 169 | 2090 | 1.34 | 1.12 | – | 1.60 | 1.34 | 1.12 | – | 1.59 |
| ^a^ Model 1: Adjusted for age (as time-scale) and stratified by birth cohort. | | | | | | | | | | |
| ^b^ Model 2: Model 1 + smoking. | | | | | | | | | | |
| ^c^ Normal and underweight (<25.0). | | | | | | | | | | |
| Missing values in BMI (n=38) and smoking (n=78) were imputed. | | | | | | | | | | |

| **Table S3.** Hazard ratios (HR) of kidney cancer and 95% confidence intervals (95% CI) according to smoking history at baseline in the Norwegian Offshore Petroleum Workers cohort followed 1999–2021 | | | | | | | | | | |
| --- | --- | --- | --- | --- | --- | --- | --- | --- | --- | --- |
| **Smoking** | **Cases** | **Non-cases** | **Model 1^a^** | | | | **Model 2^b^** | | | |
|  |  |  | **HR** | **95% CI** | | | **HR** | **95% CI** | | |
| Never | 40 | 450 | 1.00 | (reference) | | | 1.00 | (reference) | | |
| Former | 57 | 857 | 1.06 | 0.68 | – | 1.66 | 0.98 | 0.63 | – | 1.53 |
| Current <12 cigarettes per day | 33 | 355 | 1.19 | 0.71 | – | 1.97 | 1.24 | 0.75 | – | 2.06 |
| Current ≥12 cigarettes per day | 32 | 357 | 1.29 | 0.77 | – | 2.16 | 1.29 | 0.77 | – | 2.16 |
| ^a^ Model 1: Adjusted for age (as time-scale) and stratified by birth cohort. | | | | | | | | | | |
| ^b^ Model 2: Model 1 + body mass index (BMI) (continuous). | | | | | | | | | | |
| Missing values for BMI (n=38) and smoking (n=78) were imputed. | | | | | | | | | | |

**Technical description explaining the assessment of component association *C_3_***

In Table S4 below we extend the analysis of component assosiation *C_3_* by additional adjustment for individual occupational exposures of interest (Model 2A–2E). This was done to ensure that the association between work status (*W_j_*, using total employment duration) and kidney cancer incidence (*K*) was not through a pathway confounded by prior exposure (*E_j–_*_1_, using cumulative exposure up to basline) as presented in the DAG shown in Figure 1.

If work status is not related to kidney cancer other than through an association with occupational exposure, we would expect to see approximately null association in Table S4 after adjusting for prior exposure (blocking pathway confounded by prior exposure) conceptualized by pathway i.:

i. *W_j_* ← *E_j–_*_1_ → *K*

In Table S4 all models were also adjusted for BMI and smoking (*M_j_*), variables that may be measured confounders of the association between work status and kidney cancer:

ii. *W_j_* ← *M_j_* → *K*

In Models 2A–2E in Table S4, blocking the pathways i–ii, we observed only negligible changes in the HR estimates compared to Models 1 and Model 2 (Table 2, manuscript) suggesting that the association between total employment duration and kidney cancer is independent of exposure, and we interpret the association as being through the pathway conceptualized as unmeasured common causes (*U_j_*) of *W* and *K*:

iii. *W_j_* ← *U_j_* → *K*

• *j* is age,

• *E* is exposure offshore (single agent),

• *W* is work status,

• *M* is measured- and *U* is unmeasured common causes of *W* and *K*,

• *K* is kidney cancer diagnosis.

| **Table S4.** Component association C_3_ assessing the association between total employment duration (prior to baseline) and kidney cancer and, in the Norwegian Offshore Petroleum Workers cohort followed 1999–2021 | | | | | | | | | | | | | | | | | | | | | | |
| --- | --- | --- | --- | --- | --- | --- | --- | --- | --- | --- | --- | --- | --- | --- | --- | --- | --- | --- | --- | --- | --- | --- |
| **Total employment duration (years)** | **Cases** | **Non-cases** | **Model 2A**^a, b^ | | | | **Model 2B**^c^ | | | | **Model 2C**^d^ | | | | **Model 2D**^e^ | | | | **Model 2E**^f^ | | | |
|  |  |  | **HR** | **95% CI** | | | **HR** | **95% CI** | | | **HR** | **95% CI** | | | **HR** | **95% CI** | | | **HR** | **95% CI** | | |
| Q1 (>0–<6.0) | 54 | 524 | 1.00 | (reference) | | | 1.00 | (reference) | | | 1.00 | (reference) | | | 1.00 | (reference) | | | 1.00 | (reference) | | |
| Q2 (6.0–<12.2) | 47 | 505 | 1.01 | 0.66 | – | 1.55 | 0.96 | 0.62 | – | 1.50 | 1.04 | 0.67 | – | 1.60 | 1.02 | 0.64 | – | 1.60 | 1.06 | 0.69 | – | 1.62 |
| Q3 (12.2–<18.3) | 39 | 531 | 0.83 | 0.52 | – | 1.34 | 0.75 | 0.45 | – | 1.25 | 0.87 | 0.55 | – | 1.37 | 0.84 | 0.50 | – | 1.42 | 0.89 | 0.57 | – | 1.41 |
| Q4 (18.3–<33.5) | 29 | 530 | 0.59 | 0.35 | – | 0.99 | 0.51 | 0.29 | – | 0.91 | 0.64 | 0.37 | – | 1.11 | 0.60 | 0.33 | – | 1.11 | 0.67 | 0.40 | – | 1.12 |
| *P*-trend ^g^ |  |  | *0.049* |  |  |  | *0.022* |  |  |  | *0.116* |  |  |  | *0.120* |  |  |  | *0.126* |  |  |  |
| ^a^ Model 2: Cox regression adapted to the case-cohort design, stratified by birth cohort and adjusted for age (as time-scale), body mass index (BMI), and smoking. Missing values in BMI (n=38) and smoking (n=78) were imputed. | | | | | | | | | | | | | | | | | | | | | | |
| ^b^ Model 2A: Model 2 + cumulative benzene exposure (continuous). | | | | | | | | | | | | | | | | | | | | | | |
| ^c^ Model 2B: Model 2 + cumulative crude oil skin exposure (continuous). | | | | | | | | | | | | | | | | | | | | | | |
| ^d^ Model 2C: Model 2 + cumulative asbestos exposure (continuous). | | | | | | | | | | | | | | | | | | | | | | |
| ^e^ Model 2D: Model 2 + cumulative chlorinated degreasing agents (CDA) exposure (continuous). | | | | | | | | | | | | | | | | | | | | | | |
| ^f^ Model 2E: Model 2 + cumulative welding fumes exposure (continuous). | | | | | | | | | | | | | | | | | | | | | | |
| ^g^ Modelled by using the medians within each category to test for linear trend. | | | | | | | | | | | | | | | | | | | | | | |
| Abbreviations: CI, confidence interval; HR, hazard ratio; Q, quartile. | | | | | | | | | | | | | | | | | | | | | | |

**Technical description explaining the assessment of component association *C_1_***

For evaluation of component *C*_1_ in our study sample shown in Table S5 below, we followed an approach more similar to Bertke et al.(1) than that of Naimi et al.(2) We analysed the association between termination of employment and a time-varying variable representing cumulative exposure restricted to a three-year period prior to observation. As noted by Bertke et al.(1) cumulative exposure is largely dependent on duration of employment, and with the presence of a healthy worker survivor bias (HWSB) it might be more appropriate to consider an exposure metric more related to the exposure intensity experienced prior to observation. This approach should coincide with the proposed underlying mechanism of HWSB as depicted by Naimi et al.(2) presented in the DAG shown in Figure 1 as the pathway: *E_j–_*_1_ → *W_j_*.

Follow-up for this analysis started three years after first offshore employment for each worker, ending when employment was terminated, or 31. December 1996, whichever occurred first. The reference group included only workers considered unlikely exposed (workers given only 0 in JEM-score for the exposure in question). Exposure was modelled categorically by tertiles defined at termination among those who left work offshore. In the models we also adjusted for year of hire. When assessing offshore asbestos exposure, we additionally adjusted for employment before offshore work with likely asbestos exposure (pre-offshore asbestos exposure (no/yes)) recorded at baseline.

Pre-offshore asbestos exposure was defined as workers listing employment before starting work offshore within the type of work or industries defined in the questionnaire as: work in ship engine rooms (machinist), work in production industry (chemical, heavy, mechanized), building and construction, painting/surface treatment, and electrical occupations. The selection of industries was based on elevated standardized incidence ratios (SIRs) for pleural/peritoneal mesotheliomas in certain occupational categories for Norwegian workers.(3)

**References**

1 Bertke, S. J., Keil, A. P. & Daniels, R. D. Lung Cancer Mortality and Styrene Exposure in the Reinforced-Plastics Boatbuilding Industry: Evaluation of Healthy Worker Survivor Bias. *Am J Epidemiol* **190**, 1784-1792 (2021).

2 Naimi, A. I. P., Cole, S. R. P., Hudgens, M. G. P., Brookhart, M. A. P. & Richardson, D. B. P. Assessing the component associations of the healthy worker survivor bias: occupational asbestos exposure and lung cancer mortality. *Ann Epidemiol* **23**, 334-341 (2013).

3 Pukkala, E., Martinsen, J. I., Lynge, E., Gunnarsdottir, H. K., Sparén, P., Tryggvadottir, L. *et al.* Occupation and cancer - follow-up of 15 million people in five Nordic countries. *Acta Oncol* **48**, 646-790 (2009).

| **Table S5.** Component association C_1_ analyzing the association between past cumulative exposure (JEM-score x years), restricted to a three-year prior to observation, and termination of employment offshore (1968–1996) | | | | | | |
| --- | --- | --- | --- | --- | --- | --- |
| **Exposure**^b^ | **N left work** | **Person-years** | **Model**^a^ | | | |
|  |  |  | **HR** | **95% CI** | | |
| **Benzene** (ppm x years) |  |  |  |  |  |  |
| 0 | 357 | 7918.5 | 1.00 | (reference) | | |
| >0.000–<0.033 | 158 | 4579.5 | 1.05 | 0.99 | – | 1.28 |
| 0.033–<0.054 | 171 | 3774.4 | 0.98 | 0.81 | – | 1.18 |
| 0.054–<0.171 | 157 | 5256.3 | 0.70 | 0.58 | – | 0.85 |
| *P*-trend ^c^ |  |  | *<0.001* |  |  |  |
| **Crude oil skin** (JEM-score x years) |  |  |  |  |  |  |
| 0 | 298 | 5867.8 | 1.00 | (reference) | | |
| >0–<2.8 | 196 | 4899.2 | 0.98 | 0.80 | – | 1.19 |
| 2.8–<3.1 | 250 | 7173.4 | 0.75 | 0.63 | – | 0.90 |
| 3.1–<9.0 | 99 | 3588.4 | 0.58 | 0.46 | – | 0.73 |
| *P*-trend ^c^ |  |  | *<0.001* |  |  |  |
| **CDA** (JEM-score x years) |  |  |  |  |  |  |
| 0 | 191 | 1648.5 | 1.00 | (reference) | | |
| >0–<3.5 | 230 | 5845.4 | 0.91 | 0.75 | – | 1.12 |
| 3.5–<5.9 | 177 | 4512.3 | 0.98 | 0.79 | – | 1.21 |
| 5.9–<9.0 | 245 | 7018.9 | 0.87 | 0.71 | – | 1.06 |
| *P*-trend ^c^ |  |  | *0.198* |  |  |  |
| **Asbestos**^d^ (JEM-score x years) |  |  |  |  |  |  |
| 0 | 255 | 5187.9 | 1.00 | (reference) | | |
| >0–<2.7 | 145 | 4336.3 | 0.73 | 0.59 | – | 0.91 |
| 2.7–<4.4 | 299 | 7868.0 | 0.75 | 0.63 | – | 0.90 |
| 4.4–<29.4 | 144 | 4136.5 | 1.20 | 0.96 | – | 1.50 |
| *P*-trend ^c^ |  |  | *0.013* |  |  |  |
| **Welding fumes** (JEM-score x years) |  |  |  |  |  |  |
| 0 | 531 | 13264.4 | 1.00 | (reference) | | |
| >0–<1.8 | 147 | 3546.2 | 1.05 | 0.86 | – | 1.27 |
| 1.8–<3.0 | 87 | 2348.6 | 0.88 | 0.69 | – | 1.12 |
| 3.0–<9.0 | 78 | 2369.6 | 0.86 | 0.67 | – | 1.10 |
| *P*-trend ^c^ |  |  | *0.184* |  |  |  |
| ^a^ Cox regression adapted to the case-cohort design, stratified by birth cohort and adjusted for age (as time-scale) and year of first hire (continuous). | | | | | | |
| ^b^ Cumulative exposure restricted to a three-year prior to observation. | | | | | | |
| ^c^ Modelled by using the medians of each category to test for linear trend. | | | | | | |
| ^d^ Additionally adjusted for pre-offshore asbestos exposure (no/yes). | | | | | | |

| **Table S6.** Hazard ratios (HR) of kidney cancer and 95% confidence intervals (95% CI) according to average intensity of exposure in the Norwegian Offshore Petroleum Workers cohort followed 1999–2021 | | | | | | | | | | | | | | |
| --- | --- | --- | --- | --- | --- | --- | --- | --- | --- | --- | --- | --- | --- | --- |
| **Average intensity** | **Cases** | **Non-cases** | **Model 1**^a^ | | | | **Model 2**^b^ | | | | **Model 3**^c^ | | | |
|  |  |  | **HR** | **95% CI** | | | **HR** | **95% CI** | | | **HR** | **95% CI** | | |
| **Benzene** (ppm) |  |  |  |  |  |  |  |  |  |  |  |  |  |  |
| 0 | 54 | 640 | 1.00 | (reference) | | | 1.00 | (reference) | | | 1.00 | (reference) | | |
| >0.000–0.015 | 66 | 833 | 0.92 | 0.63 | – | 1.34 | 0.94 | 0.64 | – | 1.38 | 0.97 | 0.66 | – | 1.43 |
| >0.015–0.030 | 21 | 317 | 0.82 | 0.48 | – | 1.39 | 0.77 | 0.44 | – | 1.34 | 0.82 | 0.47 | – | 1.44 |
| >0.030–0.057 | 28 | 300 | 1.15 | 0.70 | – | 1.87 | 1.19 | 0.72 | – | 1.95 | 1.26 | 0.77 | – | 2.06 |
| *P*-trend ^d^ |  |  | *0.610* |  |  |  | *0.583* |  |  |  | *0.430* |  |  |  |
| **Crude oil skin** (JEM-score) |  |  |  |  |  |  |  |  |  |  |  |  |  |  |
| 0 | 38 | 505 | 1.00 | (reference) | | | 1.00 | (reference) | | | 1.00 | (reference) | | |
| >0–<1.0 | 52 | 635 | 0.84 | 0.54 | – | 1.32 | 0.90 | 0.57 | – | 1.41 | 0.94 | 0.60 | – | 1.47 |
| 1.0–<1.5 | 47 | 618 | 0.77 | 0.49 | – | 1.21 | 0.75 | 0.47 | – | 1.19 | 0.78 | 0.49 | – | 1.23 |
| 1.5–3.0 | 32 | 332 | 1.05 | 0.64 | – | 1.72 | 1.09 | 0.66 | – | 1.80 | 1.16 | 0.70 | – | 1.91 |
| *P*-trend ^d^ |  |  | *0.929* |  |  |  | *0.825* |  |  |  | *0.673* |  |  |  |
| **CDA** (JEM-score) |  |  |  |  |  |  |  |  |  |  |  |  |  |  |
| 0 | 26 | 334 | 1.00 | (reference) | | | 1.00 | (reference) | | | 1.00 | (reference) | | |
| >0–<1.27 | 48 | 601 | 0.80 | 0.48 | – | 1.34 | 0.80 | 0.48 | – | 1.36 | 0.82 | 0.49 | – | 1.38 |
| 1.27–<2.0 | 55 | 567 | 0.99 | 0.60 | – | 1.63 | 0.97 | 0.59 | – | 1.62 | 1.02 | 0.62 | – | 1.69 |
| 2.0–3.0 | 40 | 588 | 0.87 | 0.51 | – | 1.47 | 0.87 | 0.51 | – | 1.48 | 0.88 | 0.52 | – | 1.50 |
| *P*-trend ^d^ |  |  | *0.864* |  |  |  | *0.862* |  |  |  | *0.928* |  |  |  |
| **Asbestos** (JEM-score) |  |  |  |  |  |  |  |  |  |  |  |  |  |  |
| 0 | 40 | 457 | 1.00 | (reference) | | | 1.00 | (reference) | | | 1.00 | (reference) | | |
| >0–<0.9 | 52 | 558 | 0.87 | 0.56 | – | 1.35 | 0.89 | 0.57 | – | 1.38 | 0.92 | 0.59 | – | 1.43 |
| 0.9–<1.3 | 42 | 523 | 0.77 | 0.49 | – | 1.23 | 0.80 | 0.50 | – | 1.28 | 0.83 | 0.52 | – | 1.32 |
| 1.3–9.8 | 35 | 552 | 0.76 | 0.47 | – | 1.23 | 0.74 | 0.45 | – | 1.21 | 0.78 | 0.48 | – | 1.28 |
| *P*-trend ^d^ |  |  | *0.287* |  |  |  | *0.245* |  |  |  | *0.340* |  |  |  |
| **Welding fumes** (JEM-score) |  |  |  |  |  |  |  |  |  |  |  |  |  |  |
| 0 | 108 | 1157 | 1.00 | (reference) | | | 1.00 | (reference) | | | 1.00 | (reference) | | |
| >0–<0.6 | 20 | 317 | 0.67 | 0.40 | – | 1.10 | 0.70 | 0.42 | – | 1.16 | 0.70 | 0.42 | – | 1.17 |
| 0.6–<1.0 | 20 | 306 | 0.72 | 0.44 | – | 1.19 | 0.68 | 0.40 | – | 1.16 | 0.71 | 0.42 | – | 1.21 |
| 1.0–3.0 | 21 | 310 | 0.78 | 0.47 | – | 1.27 | 0.79 | 0.48 | – | 1.29 | 0.82 | 0.50 | – | 1.34 |
| *P*-trend ^d^ |  |  | *0.175* |  |  |  | *0.178* |  |  |  | *0.244* |  |  |  |
| ^a^ Model 1: Cox regression adapted to the case-cohort design, stratified birth cohort and adjusted for age (as time-scale) | | | | | | | | | | | | | | |
| ^b^ Model 2: Model 1 + body mass index and smoking. Missing values in BMI (n=38) and smoking (n=78) were imputed. | | | | | | | | | | | | | | |
| ^c^ Model 3: Model 2 + total employment duration. | | | | | | | | | | | | | | |
| ^d^ Modelled by using the medians within each category to test for linear trend. | | | | | | | | | | | | | | |

| **Table S7.** Hazard ratios (HR) of first primary renal cell carcinoma (RCC) and 95% confidence intervals (95% CI) according to total employment duration and cumulative exposure in the Norwegian Offshore Petroleum Workers cohort followed 1999–2021 | | | | | | | | | | | | | | |
| --- | --- | --- | --- | --- | --- | --- | --- | --- | --- | --- | --- | --- | --- | --- |
| **Variables** | **Cases** | **Non-cases** | **Model 1**^a^ | | | | **Model 2**^b^ | | | | **Model 3**^c^ | | | |
|  |  |  | **HR** | **95% CI** | | | **HR** | **95% CI** | | | **HR** | **95% CI** | | |
| **Total duration of employment** (years) |  |  |  |  |  |  |  |  |  |  |  |  |  |  |
| >0–<6.0 | 40 | 526 | 1.00 | (reference) | | | 1.00 | (reference) | | |  |  | | |
| 6.0–<12.2 | 36 | 510 | 1.05 | 0.65 | – | 1.69 | 1.07 | 0.66 | – | 1.73 |  |  |  |  |
| 12.2–<18.3 | 26 | 529 | 0.77 | 0.46 | – | 1.31 | 0.79 | 0.46 | – | 1.34 |  |  |  |  |
| 18.3–<33.5 | 21 | 531 | 0.66 | 0.37 | – | 1.17 | 0.63 | 0.35 | – | 1.14 |  |  |  |  |
| *P*-trend ^d^ |  |  | *0.108* |  |  |  | *0.092* |  |  |  |  |  |  |  |
| **Cumulative exposures** |  |  |  |  |  |  |  |  |  |  |  |  |  |  |
| **Benzene** (ppm x years) |  |  |  |  |  |  |  |  |  |  |  |  |  |  |
| 0 | 37 | 642 | 1.00 | (reference) | | | 1.00 | (reference) | | | 1.00 | (reference) | | |
| >0.000–<0.059 | 40 | 474 | 1.26 | 0.78 | – | 2.01 | 1.27 | 0.79 | – | 2.06 | 1.22 | 0.75 | – | 1.98 |
| 0.059–<0.219 | 22 | 491 | 0.84 | 0.48 | – | 1.45 | 0.87 | 0.50 | – | 1.53 | 0.92 | 0.52 | – | 1.63 |
| 0.219–<1.542 | 24 | 489 | 0.94 | 0.55 | – | 1.63 | 0.91 | 0.52 | – | 1.60 | 1.08 | 0.60 | – | 1.95 |
| *P*-trend ^d^ |  |  | *0.534* |  |  |  | *0.434* |  |  |  | *0.995* |  |  |  |
| **Crude oil skin** (JEM-score x years) |  |  |  |  |  |  |  |  |  |  |  |  |  |  |
| 0 | 30 | 506 | 1.00 | (reference) | | | 1.00 | (reference) | | | 1.00 | (reference) | | |
| >0–<7.4 | 34 | 527 | 0.77 | 0.45 | – | 1.30 | 0.80 | 0.47 | – | 1.36 | 0.67 | 0.39 | – | 1.16 |
| 7.4–<17.6 | 34 | 527 | 0.78 | 0.47 | – | 1.32 | 0.80 | 0.48 | – | 1.36 | 0.93 | 0.54 | – | 1.59 |
| 17.6–70.5 | 25 | 536 | 0.67 | 0.38 | – | 1.18 | 0.71 | 0.40 | – | 1.25 | 1.04 | 0.53 | – | 2.05 |
| *P*-trend ^d^ |  |  | *0.266* |  |  |  | *0.323* |  |  |  | *0.739* |  |  |  |
| **CDA** (JEM-score x years) |  |  |  |  |  |  |  |  |  |  |  |  |  |  |
| 0 | 22 | 334 | 1.00 | (reference) | | | 1.00 | (reference) | | | 1.00 | (reference) | | |
| >0–<9.2 | 43 | 578 | 0.86 | 0.49 | – | 1.52 | 0.86 | 0.49 | – | 1.53 | 0.76 | 0.41 | – | 1.39 |
| 9.2–<23.0 | 32 | 589 | 0.73 | 0.41 | – | 1.29 | 0.76 | 0.42 | – | 1.35 | 0.81 | 0.46 | – | 1.43 |
| 23.0–85.4 | 26 | 595 | 0.62 | 0.34 | – | 1.13 | 0.60 | 0.32 | – | 1.11 | 0.76 | 0.39 | – | 1.46 |
| *P*-trend ^d^ |  |  | *0.103* |  |  |  | *0.090* |  |  |  | *0.540* |  |  |  |
| **Asbestos** (JEM-score x years) ^e^ |  |  |  |  |  |  |  |  |  |  |  |  |  |  |
| 0 | 31 | 458 | 1.00 | (reference) | | | 1.00 | (reference) | | | 1.00 | (reference) | | |
| >0–<7.1 | 42 | 535 | 0.85 | 0.52 | – | 1.40 | 0.90 | 0.54 | – | 1.49 | 0.86 | 0.51 | – | 1.44 |
| 7.1–<16.7 | 31 | 546 | 0.70 | 0.41 | – | 1.20 | 0.74 | 0.43 | – | 1.27 | 0.77 | 0.45 | – | 1.31 |
| 16.7–160.0 | 19 | 557 | 0.50 | 0.27 | – | 0.90 | 0.48 | 0.26 | – | 0.89 | 0.54 | 0.28 | – | 1.05 |
| *P*-trend ^d^ |  |  | *0.016* |  |  |  | *0.013* |  |  |  | *0.074* |  |  |  |
| **Welding fumes** (JEM-score x years) |  |  |  |  |  |  |  |  |  |  |  |  |  |  |
| 0 | 1162 | 78 | 1.00 | (reference) | | | 1.00 | (reference) | | | 1.00 | (reference) | | |
| >0–<3.8 | 305 | 22 | 0.95 | 0.57 | – | 1.57 | 0.98 | 0.59 | – | 1.64 | 0.92 | 0.55 | – | 1.53 |
| 3.8–<12.5 | 319 | 11 | 0.58 | 0.30 | – | 1.11 | 0.52 | 0.26 | – | 1.05 | 0.55 | 0.27 | – | 1.10 |
| 12.5–100.5 | 310 | 12 | 0.65 | 0.35 | – | 1.22 | 0.66 | 0.35 | – | 1.25 | 0.77 | 0.39 | – | 1.49 |
| *P*-trend ^d^ |  |  | *0.122* |  |  |  | *0.123* |  |  |  | *0.296* |  |  |  |
| **Surface treatment** |  |  |  |  |  |  |  |  |  |  |  |  |  |  |
| Never | 114 | 2 037 | 1.00 | (reference) | | | 1.00 | (reference) | | | 1.00 | (reference) | | |
| Ever | 9 | 59 | 3.06 | 1.41 | – | 6.62 | 3.34 | 1.53 | – | 7.28 | 3.32 | 1.53 | – | 7.19 |
| Duration (years) |  |  |  |  |  |  |  |  |  |  |  |  |  |  |
| 0 | 114 | 2 037 | 1.00 | (reference) | | | 1.00 | (reference) | | | 1.00 | (reference) | | |
| >0–<8.1 | 6 | 28 | 4.26 | 1.64 | – | 11.1 | 4.70 | 1.81 | – | 12.2 | 4.29 | 1.66 | – | 11.1 |
| 8.1–<21.3 | 3 | 31 | 1.96 | 0.55 | – | 6.96 | 2.12 | 0.59 | – | 7.59 | 2.29 | 0.65 | – | 8.14 |
| *P*-trend ^d^ |  |  | *0.083* |  |  |  | *0.060* |  |  |  | *0.041* |  |  |  |
| ^a^ Model 1: Cox regression adapted to the case-cohort design, stratified by birth cohort and adjusted for age (as time-scale). | | | | | | | | | | | | | | |
| ^b^ Model 2: Model 1 + body mass index and smoking. Missing values in BMI (n=38) and smoking (n=78) were imputed. | | | | | | | | | | | | | | |
| ^c^ Model 3: Model 2 + total employment duration. | | | | | | | | | | | | | | |
| ^d^ Modelled by using the medians within each category to test for linear trend. | | | | | | | | | | | | | | |

| **Table S8.** Hazard ratios (HR) of first primary kidney cancer and 95% confidence intervals (95% CI) according to cumulative exposure with 10- or 20-year exposure lag, in the Norwegian Offshore Petroleum Workers cohort followed 1999–2021 | | | | | | | | | | | | | | | | | | | | |
| --- | --- | --- | --- | --- | --- | --- | --- | --- | --- | --- | --- | --- | --- | --- | --- | --- | --- | --- | --- | --- |
| **Cumulative exposure** | **Cases** | **Person- yrs.** | **Model 2**^a^ **and 10-yr lag** | | | | **Model 3**^b^ **and 10-yr lag** | | | | **Cases** | **Person- yrs.** | **Model 2**^a^ **and 20-yr lag** | | | | **Model 3**^b^ **and 20-yr lag** | | | |
|  |  |  | **HR** | **95% CI** | | | **HR** | **95% CI** | | |  |  | **HR** | **95% CI** | | | **HR** | **95% CI** | | |
| **Benzene** (ppm x years) |  |  |  |  |  |  |  |  |  |  |  |  |  |  |  |  |  |  |  |  |
| 0 | 38 | 12222.0 | 1.00 | (reference) | | | 1.00 | (reference) | | | 41 | 16261.9 | 1.00 | (reference) | | | 1.00 | (reference) | | |
| >0.000–<0.059 | 39 | 9490.5 | 1.22 | 0.75 | – | 1.96 | 1.17 | 0.73 | – | 1.89 | 44 | 9770.9 | 1.55 | 0.99 | – | 2.44 | 1.57 | 0.99 | – | 2.48 |
| 0.059–<0.219 | 23 | 8556.3 | 0.90 | 0.52 | – | 1.56 | 0.96 | 0.55 | – | 1.68 | 17 | 7494.0 | 0.84 | 0.46 | – | 1.52 | 0.98 | 0.52 | – | 1.84 |
| 0.219–<1.542 | 23 | 8002.6 | 0.91 | 0.51 | – | 1.61 | 1.10 | 0.61 | – | 1.99 | 21 | 4744.6 | 1.43 | 0.77 | – | 2.67 | 1.90 | 0.98 | – | 3.68 |
| *P*-trend ^c^ |  |  | *0.605* |  |  |  | *0.915* |  |  |  |  |  | *0.669* |  |  |  | *0.172* |  |  |  |
| **Crude oil skin** (JEM-score x years) |  |  |  |  |  |  |  |  |  |  |  |  |  |  |  |  |  |  |  |  |
| 0 | 31 | 9383.3 | 1.00 | (reference) | | | 1.00 | (reference) | | | 37 | 14136.6 | 1.00 | (reference) | | | 1.00 | (reference) | | |
| >0–<7.4 | 35 | 10639.9 | 0.80 | 0.47 | – | 1.34 | 0.69 | 0.41 | – | 1.18 | 42 | 12326.6 | 1.00 | 0.63 | – | 1.61 | 1.00 | 0.62 | – | 1.62 |
| 7.4–<17.6 | 35 | 10543.8 | 0.79 | 0.48 | – | 1.33 | 0.94 | 0.56 | – | 1.59 | 31 | 8265.5 | 0.96 | 0.57 | – | 1.62 | 1.28 | 0.71 | – | 2.31 |
| 17.6–70.5 | 22 | 7704.5 | 0.71 | 0.39 | – | 1.26 | 1.04 | 0.52 | – | 2.07 | 13 | 3542.7 | 0.91 | 0.44 | – | 1.89 | 1.44 | 0.64 | – | 3.28 |
| *P*-trend ^c^ |  |  | *0.323* |  |  |  | *0.760* |  |  |  |  |  | *0.780* |  |  |  | *0.296* |  |  |  |
| **CDA** (JEM-score x years) |  |  |  |  |  |  |  |  |  |  |  |  |  |  |  |  |  |  |  |  |
| 0 | 23 | 6664.5 | 1.00 | (reference) | | | 1.00 | (reference) | | | 30 | 11646.2 | 1.00 | (reference) | | | 1.00 | (reference) | | |
| >0–<9.2 | 42 | 11370.4 | 0.85 | 0.48 | – | 1.49 | 0.75 | 0.42 | – | 1.35 | 50 | 12797.7 | 1.18 | 0.71 |  | 1.96 | 1.18 | 0.70 | – | 1.96 |
| 9.2–<23.0 | 34 | 10875.4 | 0.79 | 0.45 | – | 1.40 | 0.87 | 0.50 | – | 1.53 | 32 | 9078.9 | 0.99 | 0.56 |  | 1.74 | 1.12 | 0.62 | – | 2.03 |
| 23.0–85.4 | 34 | 9361.1 | 0.61 | 0.33 | – | 1.14 | 0.81 | 0.41 | – | 1.58 | 11 | 4748.6 | 0.55 | 0.25 |  | 1.19 | 0.69 | 0.30 | – | 1.56 |
| *P*-trend ^c^ |  |  | *0.125* |  |  |  | *0.702* |  |  |  |  |  | *0.067* |  |  |  | *0.358* |  |  |  |
| **Asbestos** (JEM-score x years)^d^ |  |  |  |  |  |  |  |  |  |  |  |  |  |  |  |  |  |  |  |  |
| 0 | 32 | 8762 | 1.00 | (reference) | | | 1.00 | (reference) | | | 38 | 13283.6 | 1.00 | (reference) | | | 1.00 | (reference) | | |
| >0–<7.1 | 41 | 10902.8 | 0.90 | 0.55 | – | 1.47 | 0.86 | 0.52 | – | 1.42 | 51 | 11447.4 | 1.24 | 0.79 | – | 1.95 | 1.24 | 0.79 | – | 1.96 |
| 7.1–<16.7 | 32 | 9956.7 | 0.80 | 0.47 | – | 1.34 | 0.85 | 0.50 | – | 1.43 | 20 | 7644.7 | 0.67 | 0.38 | – | 1.18 | 0.72 | 0.39 | – | 1.32 |
| 16.7–160.0 | 18 | 8649.9 | 0.51 | 0.27 | – | 0.95 | 0.59 | 0.30 | – | 1.17 | 14 | 5895.7 | 0.63 | 0.32 | – | 1.23 | 0.72 | 0.35 | – | 1.46 |
| *P*-trend ^c^ |  |  | *0.025* |  |  |  | *0.149* |  |  |  |  |  | *0.047* |  |  |  | *0.194* |  |  |  |
| **Welding fumes** (JEM-score x years) |  |  |  |  |  |  |  |  |  |  |  |  |  |  |  |  |  |  |  |  |
| 0 | 79 | 21812.1 | 1.00 | (reference) | | | 1.00 | (reference) | | | 81 | 24787.8 | 1.00 | (reference) | | | 1.00 | (reference) | | |
| >0–<3.8 | 21 | 5970.5 | 0.93 | 0.56 | – | 1.56 | 0.87 | 0.52 | – | 1.46 | 22 | 5801.1 | 1.06 | 0.64 | – | 1.75 | 1.03 | 0.62 | – | 1.70 |
| 3.8–<12.5 | 10 | 5511.4 | 0.47 | 0.23 | – | 0.98 | 0.50 | 0.24 | – | 1.04 | 12 | 4856.1 | 0.66 | 0.33 | – | 1.30 | 0.73 | 0.37 | – | 1.44 |
| 12.5–100.5 | 13 | 4977.5 | 0.75 | 0.41 | – | 1.40 | 0.88 | 0.46 | – | 1.68 | 8 | 2826.4 | 0.75 | 0.35 | – | 1.62 | 0.89 | 0.40 | – | 1.97 |
| *P*-trend ^c^ |  |  | *0.219* |  |  |  | *0.480* |  |  |  |  |  | *0.290* |  |  |  | *0.595* |  |  |  |

Table S8 continues on the next page

Tab. S8 cont.

| **Surface treatment** | **Cases** | **Person- yrs.** | **Model 2**^a^ **and 10-yr lag** | | | | **Model 3**^b^ **and 10-yr lag** | | | | **Cases** | **Person- yrs.** | **Model 2^a^ and 20-yr lag** | | | | **Model 3^b^ and 20-yr lag** | | | |
| --- | --- | --- | --- | --- | --- | --- | --- | --- | --- | --- | --- | --- | --- | --- | --- | --- | --- | --- | --- | --- |
|  |  |  | **HR** | **95% CI** | | | **HR** | **95% CI** | | |  |  | **HR** | **95% CI** | | | **HR** | **95% CI** | | |
| Never | 114 | 37244.1 | 1.00 | (reference) | | | 1.00 | (reference) | | | 116 | 37505.6 | 1.00 | (reference) | | | 1.00 | (reference) | | |
| Ever | 9 | 1027.3 | 3.39 | 1.55 | – | 7.41 | 3.38 | 1.56 | – | 7.32 | 7 | 765.8 | 3.04 | 1.28 | – | 7.23 | 3.05 | 1.30 | – | 7.18 |
| Duration (years) |  |  |  |  |  |  |  |  |  |  |  |  |  |  |  |  |  |  |  |  |
| 0 | 114 | 37244.1 | 1.00 | (reference) | | | 1.00 | (reference) | | | 116 | 37505.6 | 1.00 | (reference) | | | 1.00 | (reference) | | |
| >0–<8.1 | 6 | 573.6 | 4.45 | 1.74 | – | 11.4 | 4.10 | 1.61 | – | 10.4 | 6 | 567.3 | 3.96 | 1.60 | – | 9.80 | 3.77 | 1.53 | – | 9.34 |
| 8.1–<21.3 | 3 | 453.7 | 2.30 | 0.64 | – | 8.31 | 2.51 | 0.70 | – | 8.96 | 1 | 198.5 | 1.23 | 0.15 | – | 10.2 | 1.40 | 0.17 | – | 11.6 |
| P-trend ^c^ |  |  | *0.047* |  |  |  | *0.029* |  |  |  |  |  | *0.209* |  |  |  | *0.151* |  |  |  |
| ^a^ Model 2: Cox regression adapted to the case-cohort design, stratified by birth cohort and adjusted for age (as time-scale), body mass index and smoking. | | | | | | | | | | | | | | | | | | | | |
| ^b^ Model 3: Model 2 + total employment duration. | | | | | | | | | | | | | | | | | | | | |
| ^c^ Modelled by using the medians within each category to test for linear trend. | | | | | | | | | | | | | | | | | | | | |
| Missing values in BMI (n=38) and smoking (n=78) were imputed. | | | | | | | | | | | | | | | | | | | | |
